# Clinical and prognostic features of patients with detailed RAS*/BRAF*-mutant colorectal cancer in Japan

**DOI:** 10.1186/s12885-021-08271-z

**Published:** 2021-05-07

**Authors:** Tatsuki Ikoma, Mototsugu Shimokawa, Masahito Kotaka, Toshihiko Matsumoto, Hiroki Nagai, Shogen Boku, Nobuhiro Shibata, Hisateru Yasui, Hironaga Satake

**Affiliations:** 1 Department of Medical Oncology, Kobe City Medical Center General Hospital, Kobe, 650-0047 Japan; 2 Department of Biostatistics, Yamaguchi University Graduate School of Medicine, Ube, 755-0046 Japan; 3 Department of Gastrointestinal Cancer Center, Sano Hospital, Kobe, 655-0031 Japan; 4 Cancer Treatment Center, Kansai Medical University Hospital, 2-3-1, Shinmachi, Hirakata, Osaka 573-1191 Japan

**Keywords:** Colorectal cancer, *KRAS* Exon2, *KRAS* non-Exon2, *NRAS*

## Abstract

**Background:**

RAS*/BRAF*^V600E^ mutations are the most remarkable oncogenic driver mutations in colorectal cancer (CRC) and play an important role in treatment selection. No data are available regarding the clinical and prognostic features of patients with detailed RAS*/BRAF*^V600E^-mutant metastatic CRC (mCRC) in Japan.

**Methods:**

A total of 152 chemotherapy-naïve patients with mCRC were included in this study between August 2018 and July 2019. Tumor samples were collected, and RAS*/BRAF*^V600E^ status was investigated. RAS*/BRAF*^V600E^ status was examined using a MEBGEN RASKET-B kit and polymerase chain reaction reverse sequence-specific oligonucleotide method.

**Results:**

RAS*/BRAF*^V600E^ mutations were detected in 54% of cases (*KRAS* codon 12, 26%; *KRAS* codon 13, 17%; *KRAS* non-Exon2, 5%; *NRAS*, 5%; and *BRAF*^V600E^, 7%). *BRAF*^V600E^-mutant CRC mainly existed in the right colon, whereas *KRAS* non-Exon2 and *NRAS*-mutant CRC was predominantly present in the left colon. *KRAS* non-Exon2 and *NRAS*-mutant CRC were associated with shorter survival time than RAS wild-type CRC (hazard ratio [HR], 2.26; 95% confidence interval [CI], 0.64–8.03; *p* = 0.19; HR, 2.42; 95% CI, 0.68–8.61; *p* = 0.16) and significantly shorter overall survival than *KRAS* Exon2-mutant CRC (HR, 3.88; 95% CI, 0.92–16.3; *p* = 0.04; HR, 4.80; 95% CI, 1.14–20.2; *p* = 0.02).

**Conclusions:**

In our multicenter study, the findings elucidated the clinical and prognostic features of patients with detailed RAS*/BRAF*^V600E^-mutant mCRC in Japan.

## Background

Colorectal cancer (CRC) is one of the most predominant malignant tumors worldwide, including in Japan. Treatment for advanced recurrent or metastatic CRC (mCRC) aims to control disease activity using anticancer drugs. Investigating biomarkers, especially rat sarcoma (RAS) and rapidly accelerated fibrosarcoma (RAF), are imperative for drug selection in mCRC treatment. RAS and RAF mutations control various activities, such as angiogenesis, proliferation, and apoptosis, and play an important role as prognostic and predictive indicators in CRC treatment [1–9].

Patients with mCRC with RAS (*KRAS/NRAS*) mutations receive less benefit from anti-epidermal growth factor receptor (EGFR) therapy because RAS mutations activate downstream pathways without depending on EGFR and trigger primary resistance [2, 7, 10–14]. In particular, mutations in the DNA at position 12 in the *KRAS* protein are general; the *KRAS* p.G12C mutation accounts for ~ 3% of patients with CRC and is significantly associated with poor prognosis [15]. Although there is no adequate progress in drug development for RAS-mutant tumors, recently developed drugs are expected to be effective. The CodeBreak 100 trial revealed the potent antitumor effects of AMG510, a novel *KRAS* G12C inhibitor, against *KRAS* G12C-mutant solid tumors, including mCRC [16].

Focusing on *BRAF*^V600E^, previous studies have shown that patients with mCRC with *BRAF* mutations have worse outcomes than those with *BRAF* wild-type [17–19]. There have been discrepant results regarding the efficacy of anti-EGFR antibodies in *BRAF*- and *KRAS*-mutant cases [20, 21]. In the BEACON CRC trial, a novel triplet combination regimen of encorafenib (BRAF inhibitor), binimetinib (MEK inhibitor), and cetuximab (anti-EGFR antibody), or a novel doublet combination regimen of encorafenib and cetuximab showed benefits compared with the current standard therapy and is now recognized as a new standard therapy as 2nd or later-line treatment [22].

Moreover, consideration of genetic abnormality is essential in the recent treatment of CRC, including microsatellite instability (MSI-H) and mismatch repair deficiency (dMMR). Immune checkpoint inhibitors have become the standard therapy for patients with specific factors detected for immune checkpoint inhibitors, and these inhibitors have also been shown to be very effective as 1st line treatment [23].

The presence or absence of RAS*/BRAF* mutations can affect anticancer therapy options. In general, patients with RAS*/BRAF* mutations have different clinical characteristics, and new therapeutic agents developed for driver mutations of these CRCs have been gaining popularity [19, 24]. However, no detailed data have been reported on the clinical and prognostic features in Asian patients, including those from Japan, with detailed RAS*/BRAF*^V600E^-mutant mCRC. Therefore, in the present multicenter retrospective study, we aimed to determine the clinical and prognostic features of mCRC with a detailed RAS*/BRAF*^V600E^ mutation in Japan.

## Methods

### Patient selection and characteristics

We performed a retrospective study of patients whose tissue RAS*/BRAF* testing was performed between August 2018 and July 2019 and observed them from the date of registration until July 2020. In total, 152 patients with advanced recurrent CRC were included in the present study. Patient tumor samples taken from primary or metastatic sites were used to investigate the RAS*/BRAF*^V600E^ mutation status. The following clinical data were collected from three institutions: age, sex, location of the primary tumor, pathological differentiation, stage and TNM grade, metastatic sites, first-line systemic chemotherapy regimen, duration and the best efficacy of first-line chemotherapy, date of confirmation of tumor growth after first-line chemotherapy, date of last consultation date, and date of death.

### Analysis for RAS/BRAF mutation

Genomic DNA was detected in each patient using formalin-fixed paraffin-embedded tumor samples. In total, 49 RAS*/BRAF* mutations were analyzed using the MEBGEN RASKET-B kit and polymerase chain reaction reverse sequence-specific oligonucleotide method for all enrolled cases [12, 25]. Mutations were determined using multiplex PCR and the xMAP® (Luminex®) technology. The mutations included those in *KRAS* codon 12 (G12S, G12C, G12R, G12D, G12V, and G12A), *KRAS* codon 13 (G13S, G13C, G13R, G13D, G13V, and G13A), *KRAS* codon 59 (A59T and A59G), *KRAS* codon 61 (Q61K, Q61E, Q61L, Q61P, Q61R, and Q61H), *KRAS* codon 117 (K117N), *KRAS* codon 146 (A146T, A146P, and A146V), *NRAS* codon 12 (G12S, G12C, G12R, G12D, G12V, and G12A), *NRAS* codon 13 (G13S, G13C, G13R, G13D, G13V, and G13A), *NRAS* codon 59 (A59T and A59G), *NRAS* codon 61 (Q61K, Q61E, Q61L, Q61P, Q61R, and Q61H), *NRAS* codon 117 (K117N), *NRAS* codon 146 (A146T, A146P, and A146V), and *BRAF* codon 600 (V600E).

### Assessment and statistical analysis

Disease assessment was usually performed every 8 ± 2 weeks using computed tomography (CT). The response was evaluated using CT images based on the Response Evaluation Criteria in Solid Tumors version 1.1. We defined overall survival (OS) as the time from enrollment in our study to the date of death for any reason. Patients who were alive were censored at the last follow-up. Progression-free survival (PFS) was defined as the time from enrollment in our study to initial disease progression or death, whichever occurred earlier. We defined the overall response rate as the percentage of patients who achieved a complete response or partial response relative to the total number of enrolled patients based on CT images. Statistical analyses were performed using SPSS statistics version 27.0, and a statistically significant difference was considered at a value of *p* < 0.05. Fisher’s exact test was used to compare the patient characteristics. Statistical analyses of OS and PFS were performed using the Kaplan–Meier method. The log-rank test was used to compare each group, whereas Cox regression analysis was used to estimate the hazard ratio (HR) with a 95% confidence interval (CI). We also evaluated whether RAS*/BRAF*^V600E^ status was associated with OS and PFS.

## Results

### Patient characteristics and frequency of RAS/BRAF ^V600E^ mutation subtypes

**Fig. 1:**
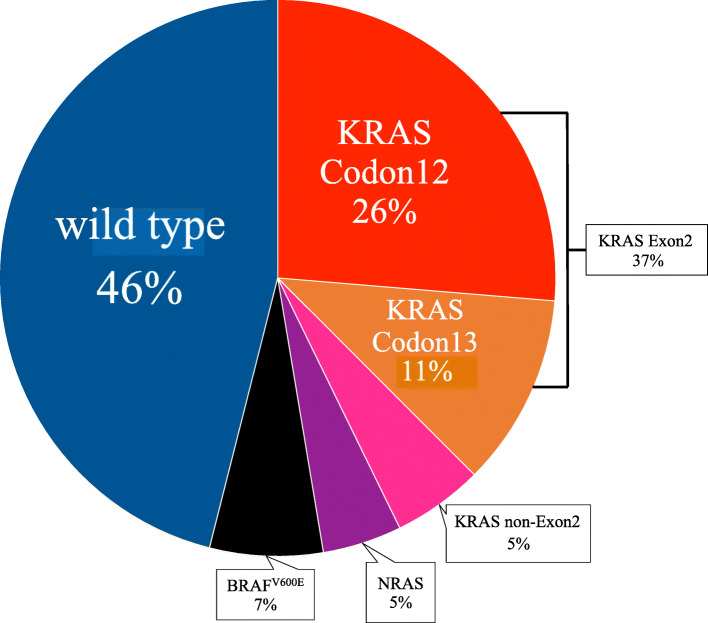
Frequencies of RAS*/BRAF*^V600E^ mutation subtypes. *N* = 152 (*KRAS* Exon2 group: *KRAS* codon 12 and codon 13; *KRAS* non-Exon2 group: other *KRAS* mutations; wild group: no mutations)

**Fig. 2:**
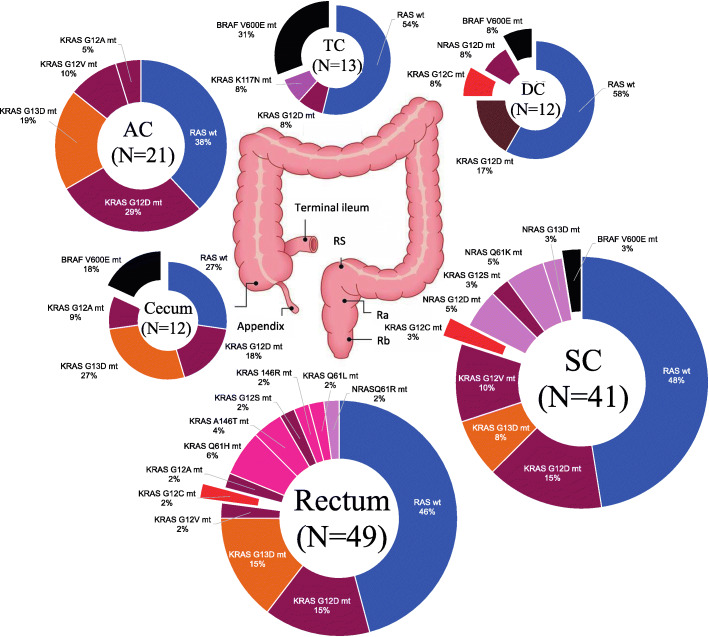
Frequency of RAS*/BRAF*^V600E^ mutations by primary tumor site (AC, ascending colon; TC, transverse colon; DC, descending colon; SC, sigmoid colon)

In total, 152 patients were investigated for RAS*/BRAF*^V600E^ status from three institutions, and the median observation period was 378 days for censored cases (range, 46–2067 days). Table 1 shows the characteristics of the patients included in this study. Patients diagnosed with stage I to III disease were those who relapsed during the observation period and were enrolled in the study. The frequency of RAS mutations was 47% (*n* = 72), whereas that of the wild-type and *BRAF*^V600E^ mutations was 46% (*n* = 70) and 7% (*n* = 10), respectively. *KRAS* mutations were found in codon 12 in 26% of cases and codon 13 in 11% of cases; therefore, we designated *KRAS* codons 12 and 13 as the *KRAS* Exon2 mutation groups. The other *KRAS* (non*-KRAS* codon 12 and non-KRAS codon 13) mutations were designated as the *KRAS* non-Exon2 mutation group, which included 5% of cases (*N* = 7; Fig. 1). The locations of the primary tumors in each RAS*/BRAF* mutation are shown in Fig. 2.

**Table 1:**
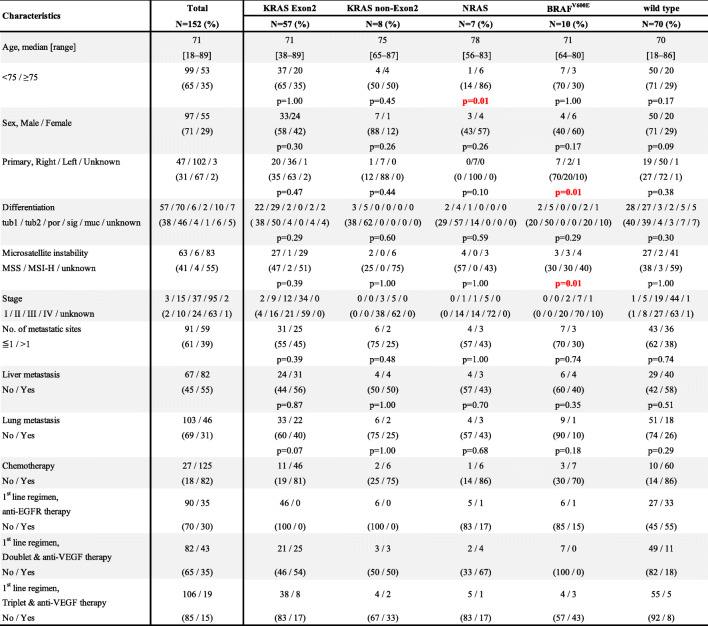
Clinical characteristics and concomitant mutations of patients with RAS*/BRAF*^V600E^ mutant colorectal cancer

*tub1/tub2* tubular adenocarcinoma, *por* poorly differentiated adenocarcinoma, *sig* signet-ring cell carcinoma, *muc* mucinous adenocarcinoma, *MSS* Microsatellite stable, *MSI-H* Microsatellite instability-high, *EGFR* Epidermal growth factor receptor, *VEGF* Vascular endothelial growth factor, *HR* Hazard ratio; *P*-value: Fisher’s exact test, and the value of the comparison between this group and other groups

### Clinicopathological characteristics of each RAS/BRAF ^V600E^ group

We investigated the relationship between RAS*/BRAF*^V600E^ mutation rate and age (< 75 and ≥ 75 years), sex, the location of the primary tumor, number of metastatic sites, liver metastasis, and lung metastasis (Table 1). *NRAS* mutations were more common in patients aged ≥75 years, whereas no correlation was observed between age and frequency in the other groups. *KRAS* non-Exon2 and *NRAS* mutations were predominantly present in the left colon, whereas *BRAF*^V600E^ mutations were significantly more common in the right colon than in the left colon (*p* = 0.01). The MSI-H group was more common in *BRAF*^V600E^ mutations (p = 0.01). We found no significant differences in other categories between the groups.

### OS of patients with each RAS/BRAF ^V600E^ status

**Fig. 3:**
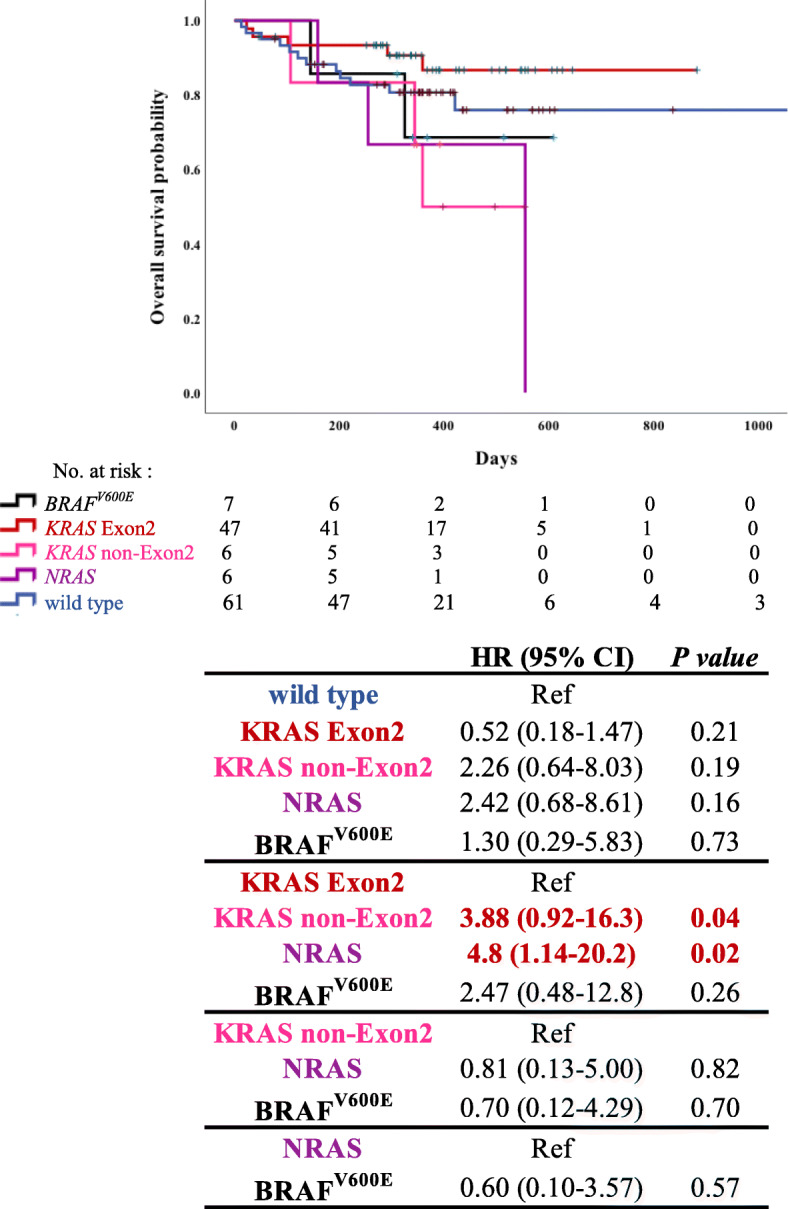
Overall survival (OS) of patients with different subtypes of RAS*/BRAF*^V600E^ mutations. Analysis of hazard ratio of OS based on RAS*/BRAF*^V600E^ mutation status in patients with colorectal cancer using Cox regression analysis (*N* = 152). *P*-value: Log-rank analysis

Among the 152 patients, 125 received systemic chemotherapy and were investigated for OS using the Kaplan-Meier method. The details of the patients are presented in Table 1. We analyzed the OS in each RAS*/BRAF*^V600E^ mutation group (Fig. 3). The OS in the wild-type group was longer than that in the *KRAS* non-Exon2, *NRAS*, and *BRAF*^V600E^ mutation groups; however, we did not observe significant differences between these groups (HR, 2.26; 95% CI, 0.64–8.03; *p* = 0.19, HR, 2.42; 95% CI, 0.68–8.61; *p* = 0.16; HR, 1.30; 95% CI, 0.29–5.83; *p* = 0.73, respectively). The OS was significantly longer in the *KRAS* Exon2 mutation group than in the *KRAS* non-Exon2 and *NRAS* mutation group (HR, 3.88; 95% CI, 0.92–16.3; *p* = 0.04; HR, 4.80; 95% CI, 1.14–20.2; *p* = 0.02). At the time of this analysis, the combination of encorafenib + binimetinib + cetuximab or encorafenib + cetuximab for the *BRAF*^V600E^ mutant CRC had not been approved; therefore, no patients were treated with these combinations.

We conducted the analysis with sex, age (< 75 and ≥ 75 years), location of the primary tumor, number of metastatic sites, liver metastasis, and lung metastasis (Table 2). As shown, none of the categories showed any apparent significant differences.

**Table 2:**
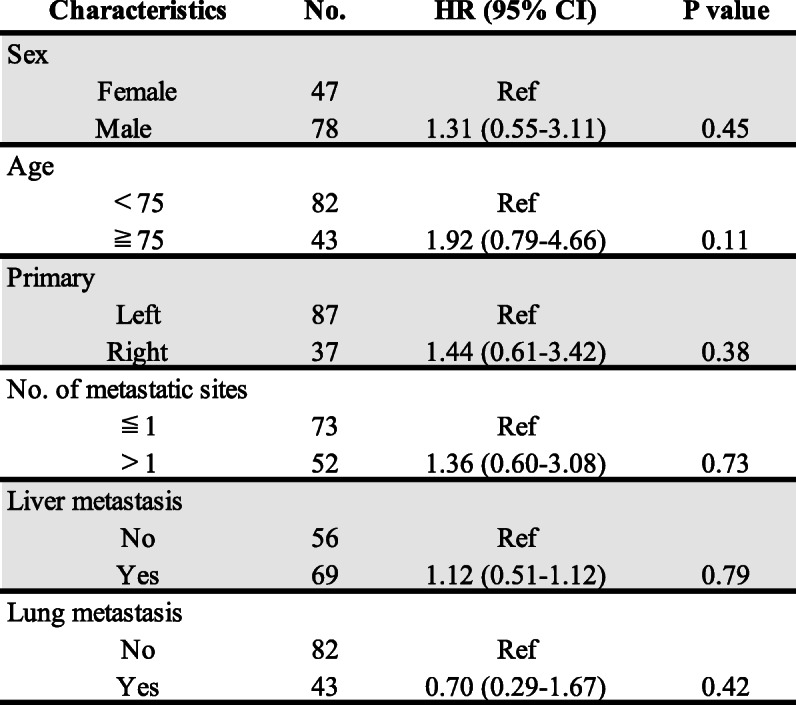
Evaluation of clinicopathological characteristics in the subgroup analysis of OS

*Ref* Reference; *P*-value: Fisher’s exact test, and the value of the comparison between this group and other groups

### OS and PFS in the patients treated with doublet therapy with anti-VEGF agents

**Fig. 4:**
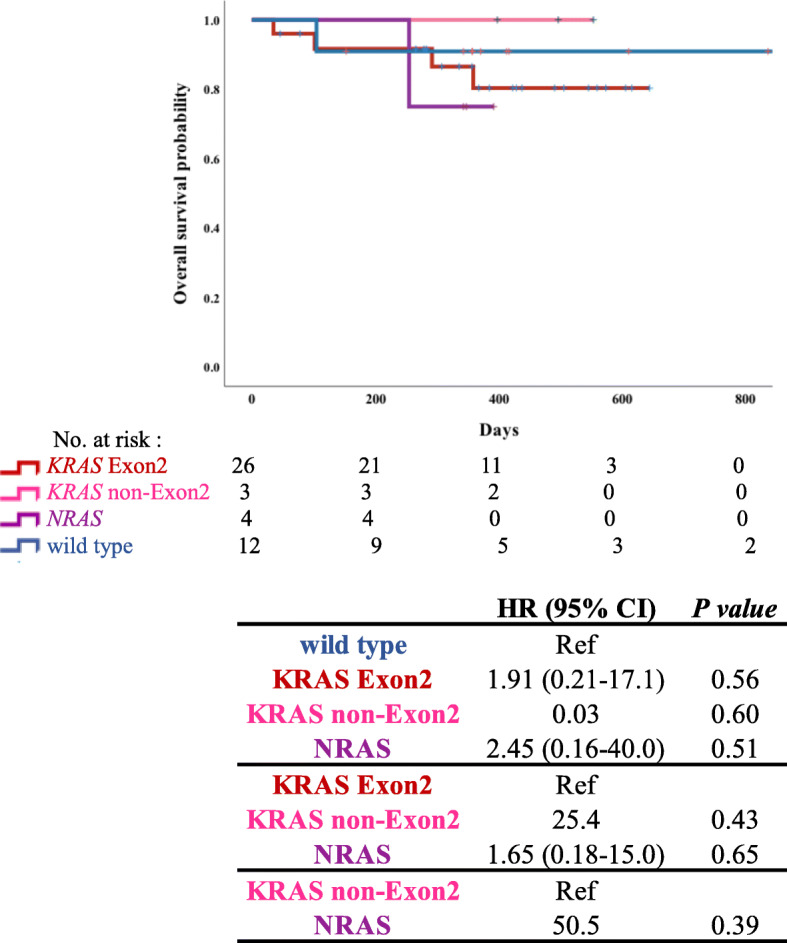
Overall survival (OS) of patients treated with doublet therapy with anti-vascular endothelial growth factor (anti-VEGF) agents. Analysis of hazard ratio of OS based on RAS mutation status in patients with colorectal cancer using Cox regression analysis (*N* = 43). P-value: Log-rank analysis

**Fig. 5:**
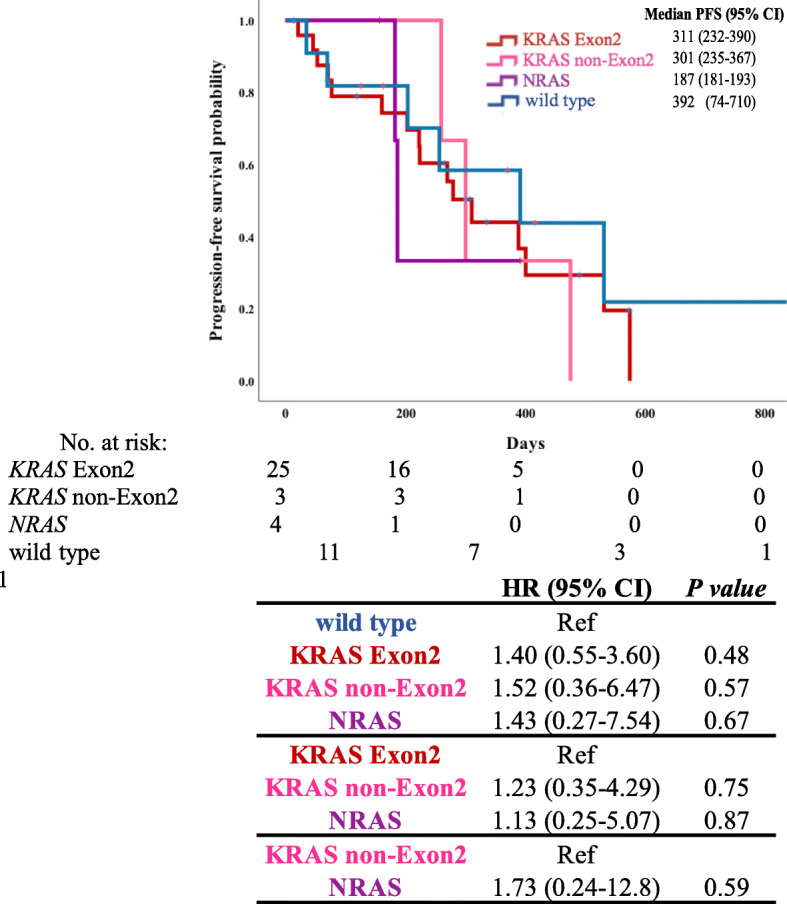
Progression-free survival (PFS) of patients treated with doublet therapy with anti-VEGF agents. Analysis of PFS of hazard ratio based on *RAS* mutation status in patients with colorectal cancer treated with doublet therapy with anti-VEGF agents using Cox regression analysis (*N* = 43). *P*-value: Log-rank analysis

Among the 125 patients who received systemic chemotherapy, 43 were treated with doublet therapy with anti-vascular endothelial growth factor (anti-VEGF) agents as primary treatment. To adjust for the treatment background, OS and PFS were evaluated only in this subgroup, excluding those who were treated with anti-EGFR antibodies against RAS wild-type. We investigated OS and PFS using the Kaplan-Meier method (Figs. 4 and 5). In the wild-type group, OS was longer than in the *KRAS* Exon2 and *NRAS* groups, and PFS was longer than that in the other groups. The median PFS was shorter in the *NRAS* group than in the other groups (187 days; 95% CI, 181–193 days).

## Discussion

In the present study, we investigated the frequency of RAS*/BRAF*^V600E^ mutations in 152 patients with mCRC in Japan and evaluated the association between each mutation and its clinical and pathological characteristics. We divided RAS mutations into the following three groups: *KRAS* codon 12 and *KRAS* codon 13 mutations as the *KRAS* Exon2 mutation group, *KRAS* mutations as the *KRAS* non-Exon2 mutation group, and *NRAS* mutation group. *KRAS* codon 12 and *KRAS* codon 13 mutations were observed in 26 and 11% of cases, respectively. In Japan, *KRAS* codon 12 and *KRAS* codon 13 mutations account for 29.9–34.1% and 3.8–7.7% of cases, respectively, which is consistent with the results of the present study [24, 26]. *NRAS* mutations have been reported in 2.5 –7.2% of cases, and in our study, *NRAS* mutations were detected in 5% of cases; thus, the frequency was generally consistent with previous reports [27]. *BRAF*^V600E^ accounted for approximately 7% of cases in the present study, whereas previous studies have reported a range of 5–21% for the *BRAF*^V600E^ mutation [18, 20]. The frequency of *KRAS* non-Exon2-mutant mCRC was the same as that in a previous study [25]; however, to date, no data have been published on the frequency of *KRAS* non-Exon2-mutant CRC in Asian populations, including Japanese patients.

We observed a relationship between the location of the primary lesion and distribution of RAS*/BRAF*^V600E^ status. Some studies have reported that *BRAF*^V600E^ mutations are more common in women over 60 years of age and in the right colon [9, 18, 19, 22]. In the present study, *BRAF*^V600E^ mutations were also more common in the right colon, with statistical significance, and MSI-H patients more commonly contained the mutations. With regard to age and sex, we did not obtain the same results as those previously reported, although it tended to be higher in females than in males. *KRAS* non-Exon2 mutations were more common in the primary tumor of the left colon. Moreover, the frequency of *KRAS* non-Exon2 mutation gradually increased and the frequency of *KRAS* Exon2 mutation decreased, as the primary location was moved from the cecum to the rectum. However, due to the small number of cases, the statistical significance could not be demonstrated as a continuum model as reported [27–29]. There are many studies on the clinical findings of *KRAS* Exon2 mutations and RAS*/BRAF*^V600E^-wild types. However, literature focusing on *KRAS* non-Exon2 mutation is rare, and the present study may be representative of *KRAS* non-Exon2 mutation. When focusing on the *NRAS* mutation, it was more commonly detected in the elderly, with a significant difference; this was also consistent with a previous report [30].

We also investigated the relationship between prognosis and RAS*/BRAF*^V600E^ status. In general, the prognosis of wild-type RAS was better than that of the RAS*/BRAF*^V600E^ mutant; however, there were no significant differences in our study. Among the RAS mutations, *KRAS* Exon2 mutation was associated with the best prognosis. *KRAS* non-Exon2 and *NRAS* mutation was associated with a shorter OS than the wild type (HR, 2.26; 95% CI, 0.64–8.03; *p* = 0.19; HR, 2.42; 95% CI, 0.68–8.61; *p* = 0.16). Furthermore, patients with *KRAS* non-Exon2 and *NRAS* mutation had a significantly shorter OS than those with *KRAS* Exon2 mutation (HR, 3.88; 95% CI, 0.92–16.3; *p* = 0.04; HR, 4.80; 95% CI, 1.14–20.2; *p* = 0.02). It has been reported that *NRAS* mutation may be associated with better prognosis compared to *KRAS* mutation [30, 31]. We considered that the results were not consistent with those previously reported because of the small number of cases in this study. The classification of *KRAS* non-Exon2 mutation has not been widely reported; however, we hypothesized that this result could be supported by the differences in OS between patients with different *KRAS* mutations. In addition, multivariate analysis of OS in all patients was performed, but no significant differences were found.

Similarly, we examined PFS for each RAS mutation type. PFS was limited to primary treatment, and the treatment regimen was limited to doublet therapy with anti-VEGF agents. Doublet therapy was defined as oxaliplatin-based and irinotecan-based regimens, and the anti-VEGF agents included bevacizumab, ramucirumab, and aflibercept. Under these conditions, *NRAS* mutation tended to be associated with shorter PFS than *KRAS* mutations; however, there were no statistically significant differences between these groups. In the RAS mutation group, there were no signs of clear superiority or inferiority, although the regimen was limited in this study. Considering the present study, *KRAS* non-Exon2 mutation had a poor prognosis. Therefore, it is recommended that *KRAS* non-Exon2 mutation should be introduced from the first chemotherapy in patients who can be treated with potent therapy such as triplet therapy with anti-VEGF agents. It is hoped that this study on RAS mutation drugs other than *KRAS* G12C will contribute to future studies in the field.

Several limitations of this research warrant mention. First, this was a retrospective study with a relatively small sample size. Second, we did not follow up with most patients until death; therefore, follow-up data were insufficient.

## Conclusion

This multicenter study revealed the detailed clinical and prognostic features of patients with RAS*/BRAF*^V600E^-mutant mCRC in Japan; each mutation had a different character. In the present study, the *KRAS* non-Exon2 and *NRAS* mutants were primarily in the left colon; to the best of our knowledge, this is the first study to reveal such findings in a Japanese population. The prognosis of patients in the *KRAS* non-Exon2 and *NRAS* mutation groups was worse than that of patients in the *KRAS* Exon2 group. Although the present study involved a relatively small number of patients, the results provide a basis for the development of specific drugs for RAS mutants, which have a poor prognosis.

## Data Availability

The datasets generated and/or analyzed during the current study are not publicly available, but are available from the corresponding author on reasonable request.

## Abbreviations

CRC: Colorectal cancer
EGFR: Epidermal growth factor receptor
mCRC: Metastatic colorectal cancer
CI: Confidence interval
CT: Computed tomography
RAF: Rapidly accelerated fibrosarcoma
RAS: Rat sarcoma
MSS: Microsatellite stable
MSI-H: Microsatellite instability-high
VEGF: Vascular endothelial growth factor
HR: Hazard ratio
dMMR: Mismatch repair deficiency
PFS: Progression-free survival
OS: Overall survival
